# Insights into the Binding of Receptor-Binding Domain (RBD) of SARS-CoV-2 Wild Type and B.1.620 Variant with hACE2 Using Molecular Docking and Simulation Approaches

**DOI:** 10.3390/biology10121310

**Published:** 2021-12-10

**Authors:** Ziyad Tariq Muhseen, Salim Kadhim, Yahiya Ibrahim Yahiya, Eid A. Alatawi, Faris F. Aba Alkhayl, Ahmad Almatroudi

**Affiliations:** 1 Department of Biomedical Engineering, College of Engineering and Applied Sciences, Nanjing University, Nanjing 210093, China; 2 School of Life Sciences, Shaanxi Normal University, Xi’an 710062, China; 3 Department of Pharmacology, College of Pharmacy, University of Alkafeel, Najaf 61001, Iraq; sfk8@leicester.ac.uk (S.K.); yahia.alkhazaily@alkafeel.edu.iq (Y.I.Y.); 4 Department of Medical Laboratory Technology, Faculty of Applied Medical Sciences, University of Tabuk, Tabuk 71491, Saudi Arabia; eid.alatawi@ut.edu.sa; 5 Department of Medical Laboratories, College of Applied Medical Sciences, Qassim University, Buraydah 51452, Saudi Arabia; Ffabaalkhiel@qu.edu.sa; 6 Department of Pharmaceutical Chemistry and Pharmacognosy, College of Dentistry and Pharmacy, Buraydah Colleges, Buraydah 51418, Saudi Arabia

**Keywords:** SARS-CoV-2, variants, B.1.620, docking, simulation, free energy

## Abstract

**Simple Summary:**

The current study provides an insight into the binding and dynamic differences between wild-type RBD and B.1.620, which harbor S477N-E484K mutations in the spike protein’s receptor-binding domain (RBD). Our analysis revealed that though the number of hydrogen bonds and salt bridges remained the same, the binding affinity of B.1.620 for ACE2 was higher than that of the wild type, consequently increasing infectivity. Moreover, the stable dynamics and other features further justify the findings, corroborating the previous literature.

**Abstract:**

Recently, a new variant, B.1620, with mutations (S477N-E484K) in the spike protein’s receptor-binding domain (RBD) has been reported in Europe. In order to design therapeutic strategies suitable for B.1.620, further studies are required. A detailed investigation of the structural features and variations caused by these substitutions, that is, a molecular level investigation, is essential to uncover the role of these changes. To determine whether and how the binding affinity of ACE2–RBD is affected, we used protein–protein docking and all-atom simulation approaches. Our analysis revealed that B.1.620 binds more strongly than the wild type and alters the hydrogen bonding network. The docking score for the wild type was reported to be −122.6 +/− 0.7 kcal/mol, while for B.1.620, the docking score was −124.9 +/− 3.8 kcal/mol. A comparative binding investigation showed that the wild-type complex has 11 hydrogen bonds and one salt bridge, while the B.1.620 complex has 14 hydrogen bonds and one salt bridge, among which most of the interactions are preserved between the wild type and B.1.620. A dynamic analysis of the two complexes revealed stable dynamics, which corroborated the global stability trend, compactness, and flexibility of the three essential loops, providing a better conformational optimization opportunity and binding. Furthermore, binding free energy revealed that the wild type had a total binding energy of −51.14 kcal/mol, while for B.1.628, the total binding energy was −68.25 kcal/mol. The current findings based on protein complex modeling and bio-simulation methods revealed the atomic features of the B.1.620 variant harboring S477N and E484K mutations in the RBD and the basis for infectivity. In conclusion, the current study presents distinguishing features of B.1.620, which can be used to design structure-based drugs against the B.1.620 variant.

## 1. Introduction

As coronaviruses continue to emerge at numerous intervals and spread at staggering levels around the world, they fall into four genera, namely, α, β, γ, and δ, in the Orthocoronavirinae subfamily of the family *Coronaviridae* [1,2,3]. Previously, the worldwide effects of beta coronaviruses have been detrimental to public health, society, and economics as reported in 2003, 2012, and 2019, respectively [4,5]. Approximately 264 million people have been infected, and 5.32 million deaths have been reported since the start of the current pandemic. In comparison, the SARS-CoV-2 case fatality ratio (CFR) is 3%, which is less than that of SARS at 10% and MERS at 35% [6,7,8,9]. However, the rapid spread of SARS-CoV-2 and the appearance of novel variants pose an increased risk to human health. The spread of β-coronaviruses among humans has been associated with these epidemics [5,6,7]. Genome sequencing insights have shown the nucleotide substitution rate as being ~1 × 10^−3^ per year for SARS-CoV-2 [10,11]. Since the outbreak of SARS-CoV-2 in late 2019, more infectious and virulent strains have been discovered, including B.1.1.7 (United Kingdom), B.1.135 (South Africa), and P.1 (Brazil). As a consequence, infectivity and hospitalization have increased. Variations in different proteins of SARS-CoV-2, particularly the spike glycoprotein, lead to a drift in the antigenicity of vaccines or other therapeutics [12,13,14,15]. The US government’s SARS-CoV-2 Interagency Group (SIG) and the European Center for Disease Prevention and Control (ECDC) have classified SARS-CoV-2 variants into three groups, i.e., variants of concern (VOCs), variants of interest (VOIs), and variants of high consequence (VOHCs) [16]. Due to the phenomenal spread of VOCs, they remained a concern because of their enhanced transmission, causing more severe disease, a significant decline in antibody neutralization, and decreased treatment effectiveness [17]. Single amino acid substitution in protein sequencing results in structural changes affecting a protein’s function. The substitution of D614 for G614 in the spike glycoprotein causes changes in the conformation of the cleavage site loop, leading to more effective S1 and S2 cleavage by enhancing furin accessibility [18]. As a result, viruses are capable of more effective transmission and replication. Globally, most SARS-CoV-2 isolates have the D614G mutation [19].

To date, many variants have been reported, among which the VOC Delta (δ)+ (AY.1 or lineage B.1.617.2.1), which evolved from Delta, demonstrated a different mutational landscape by acquiring L452R and T478K mutations in the RBD. In contrast, the δ+ variant acquired an additional mutation, the K417N mutation, alongside the L452R and T478K mutations [20]. In January 2021, the δ variant was discovered in Colombia and was reported to increase COVID-19 cases. This variant harbors E484K, N501Y, and P681H mutations in the spike protein, while many other new mutations accompany these mutations, including R346K, Y144T, Y145S, and 146N insertion [21]. Moreover, a novel VOI termed as C.37, or the Lambda/λ variant, reported in Peru with mutations L452Q and F490S in the RBD was suspected to be associated with decreased antibody neutralization susceptibility, particularly due to the F490S mutation in the RBD [22,23]. The Kappa (κ) variant, also known as B.1.617.1, first identified in India, and designated as a VOI, owns a single mutation, that is, L452R, which was suspected to be associated with reduced antibody neutralization by disrupting the respective conformational epitopes [24]. Another VOI known as Iota (ι) from lineage B.1.526, reported in New York City in early 2021, had the E484K mutation reported in the P.1 variant and was reported to partially or wholly escape the response from the two currently used therapeutic monoclonal antibodies (mAbs) and is less susceptible to neutralization [25]. The E484K substitution in the P.1 variant has been reported to establish direct interaction with the host receptor hACE2 [26]. A novel variant, C.12, has recently been reported in South Africa, but, at present, no associated risk has been confirmed. It is currently included in variants under monitoring [27].

In order to control COVID-19, multi-omics data must be collected to help understand its proteome, which is composed of 16 non-structural proteins (NSP1–NSP16) and 4 structural proteins (S, E, N, and M) [28,29]. These structural proteins accomplish a variety of functions, including adherence to the ACE2 receptor, transcriptional regulation, and replication [30]. In SARS-CoV-2, the spike protein connects with the host ACE2, which allows the virus to attach and invade the host cell [30]. Two subunits of SARS-CoV-2 (S1 and S2) enable its transmission [31]. On infection, these subunits trigger the host’s immune system and provide an optimal means of innate immunity [32]. During infection, the host cell protease cleaves the S protein at the S1/S2 cleavage site. This priming (cleavage of S protein) results in the division of protein into S1-ectoderm at the N-terminal and S2 membrane-anchored protein at the C-terminal. The former recognizes the associated cell surface receptor, and the latter is related to viral entry. The SARS-CoV S protein has conserved 14 aa RBD, which functions to recognize ACE2 and can infect both humans and bats. In these conserved 14 aa in SARS-CoV, 8 residues are highly conserved in SARS-CoV-2, supporting the assumption that ACE2 is also the receptor of this new virus [33]. In human CoVs, which are less pathogenic, the S2 cleavage site contains a monobasic sequence with no basic residues at either P2 or P4 needed for permitting furin cleavages, signifying fewer effective cleavages or sealing the initial step depending on the relevant proteases to the target cell. Although this process is believed to be the vital step for the activation of the S protein, the associated protease is still not identified [34]. For this reason, inhibiting the ACE2–RBD complex is essential to stop the spread of virulence instigated by SARS-CoV-2 [35].

Meanwhile, the use of neutralizing monoclonal antibodies (mAbs), which bind to the RBD region of the spike protein, has received greater attention but is accompanied by the risk of virus-induced resistance. As an alternative, antibodies that target sites other than the RBD and that can be used in combination may neutralize the virus more efficiently. The 4A8 antibody, reported in a recent study, neutralizes the spike protein that is isolated from the serum plasma of COVID-19 patients. 4A8 has been reported as one of the ten naturally produced antibodies that bind robustly to NTD and protect against viral infection [36]. Many small drug molecules, such as chloroquine, hydroxychloroquine, azithromycin, remdesivir, lopinavir, favipiravir, ritonavir, ribavirin, and ivermectin, are available as a treatment option for COVID-19 [35]. However, immunoglobulin, corticosteroids, interferons, tocilizumab, and many vaccines (including Sinopharm, Pfizer, AstraZeneca, Sinovac, Moderna, and Johnson Johnson) are available, but their efficacy is limited by the emergence of different variants [35]. There is a need for randomized control trials involving the whole world population to govern the effectiveness and potency of these existing possible treatment choices [37]. Because of these findings, the spike protein has the potential to be a viable drug target for therapeutic development against SARS-CoV-2 [38].

Recently, a new variant, B.1620, with mutations (S477N-E484K) in the RBD of the spike protein has been reported in Europe. It has been reported that B.1.620 is likely to escape the antibody response [39]. Most of the mutations reported in this strain are uncharacterized; however, it has been reported that the N-terminal mutations interfere with glycan binding and have been reported to partially escape natural antibodies [23,40]. A report published in Cell reported that these mutations (S477N-E484K) in the RBD increase the binding affinity for the host receptor ACE2 [41]. The mutations are reported to occur on the same loop, thus providing an opportunity for better conformational optimization and binding [42]. Mutational alterations in amino acids are anticipated to influence the structure and function of the related proteins. Therefore, it is imperative to discover the mutational landscape while creating novel antiviral therapies. Hence, it is important to determine the mutations that have been observed in the spike protein and the subsequent influence on protein structure and interaction with the host body. To design therapeutic strategies suitable for B.1.620, further studies are required. A detailed investigation of the structural features and variations caused by these substitutions, that is, a molecular level investigation, is essential to uncover the role of these changes. To determine whether and how the binding affinity of ACE2–RBD is affected, we used protein–protein docking and all-atom simulation approaches. We studied variations in the hydrogen bonding network of the wild-type and B.1.620 complexes and revealed the distinguishing features. The current study provides a basis for understanding the higher infectivity caused by B.1.620 and structure-based drug discovery against the new variants.

## 2. Material and Methods

### 2.1. Structure Retrieval and Mutants’ Modeling

Using UniProt accession number (P0DTC2), the amino acid sequence of the spike RBD of SARS-CoV-2 was retrieved, corresponding to positions 319–541. Residues S477 and E484 were mutated to Asn and Lys. The mutated sequence was then subjected to structural modeling using Modeller version v13.0 [43]. The modeled structure was refined and validated.

### 2.2. Modeling the RBD and ACE2 Complexes through Docking

Using HADDOCK, the wild-type and B.1.620 complexes (S477N-E484K) were subjected to interaction modeling to explore binding differences [44]. The interface residues were specified as reported previously, and restrained docking was performed [31]. For the wild type, the docking structures were provided by Professor Dong-Qing Wei [26]. Guru platform was used for the docking, as it employs all the available structural features to model the protein complexes and is regarded as the best feature docking. The PDBsum server [45] was utilized to discern the electrostatic interactions, hydrogen bonds, and salt bridges.

### 2.3. Dynamics of the Wild-Type and B.1.620 Complexes

The wild-type RBD–ACE2 and B.1.620 RBD–ACE2 complexes were studied at the atomic level using force field FF20SB in AMBER20 simulation package [46]. The systems were solvated by adding a water box (TIP3P) and neutralized by adding sodium ions. For each complex, two steps of energy minimization were used, 12,000 and 6000 each, using the conjugate gradient and steepest descent methods to remove any bad clashes and to relax the complexes. The heating of each complex using default parameters of 300 °K for 200 ps was performed. A 200 ns MD was accomplished for each complex using constant pressure equilibration for density equilibration for 2 ns with weak restraint. A Langevin thermostat with a 1 atm pressure and 300 °K was used to control the temperature. For the evaluation of long-range interactions, we used the particle mesh Ewald algorithm with a cutoff distance of 10 Å. The SHAKE algorithm was employed to treat covalent interactions involving hydrogen [32].

### 2.4. Post-Simulation Trajectory Analysis

Using CPPTRAJ and PTRAJ [47], structural/dynamic features were explored to determine how the recently evolved variant affects stability, flexibility, compactness, the bonding network, SASA, and protein dynamics. The stability of each complex was evaluated as the RMSD, while the residual flexibility was measured as the RMSF. The Rg and hydrogen bonds for the whole simulation trajectories were calculated to reveal structural compactness.

### 2.5. Binding Energy Differences Estimation

We used 10,000 frames from the simulation trajectory to compute the Gibbs free energy in order to explore the binding differences produced by heterogeneity in the protein structure after mutations. For each complex, the electrostatic, van der Waal, and total binding energies were calculated with the MM/GBSA method [48]. These methods are widely used and have been shown to be quite accurate [49,50,51,52,53]. Each aforementioned energy term was calculated as part of the total binding free energy.

“∆G(bind)=∆G(com)−[∆G(rec)+∆G(lig)]”

The equation below was used to calculate each energy term of the total free energy:

$$“G=G_{bond}+G_{ele\;}+G_{vdW\;}+G_{pol}+G_{npol}”$$

“*G_bond_*”, “*G_ele_*”, and “*G_vdW_*” symbolize the bonded, electrostatic, and *vdW* interactions, while “*G_pol_*” and “*G_npol_*” denote both the polar and non-polar terms.

## 3. Results and Discussion

### 3.1. Structural Modeling and Analysis

**Figure 1:**
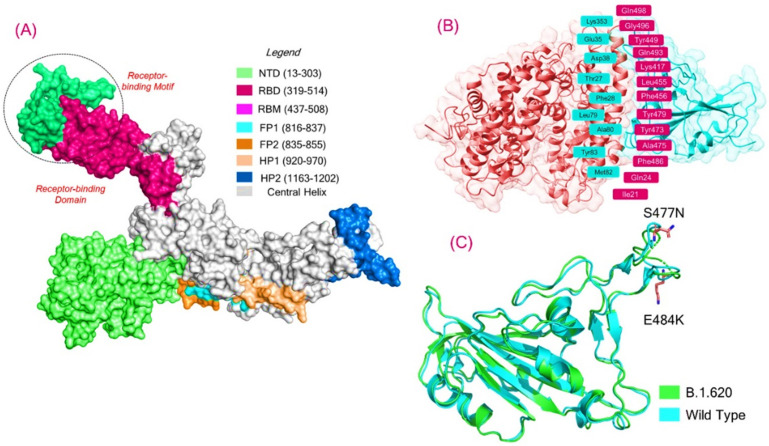
(**A**) Structural organization of the multi-domain of the spike protein. (**B**) The interface residues of ACE2–RBD complex reported experimentally are shown, while (**C**) represents the superimposed structure of the wild-type RBD and B.1.620 RBD, which revealed an RMSD difference of 0.841 Å. In the RBD domain, the two mutations, S477N and E484K, are also shown as sticks in panel C. Green represents the B.1.620 RBD, while cyan represents the wild-type RBD.

The prolonged pandemic caused by SARS-CoV-2 reported in late 2019 further exasperated the situation with the advent of new variants. The reported variants, including P.1, B.1.1.7, B.1.617, B.1.351, and B.1.618, exhibited mutations in the RBD domain of the spike protein [35]. The spike protein is an important druggable and neutralization target for vaccines [54]. Because of its prime role in binding and infection, the spike protein is deemed as an important drug target in the SARS-CoV-2 proteome. Due to continuous exposure to therapeutics, the spike protein is prone to mutations. Due to this fact, the spike protein continuously harbors mutations and evolves with more devastating effects. The spike-specific mutations, particularly the RBD, help the virus to increase binding and infectivity and evade the immune response. The spike protein, a homotrimeric complex, binds to the ACE2 protein via the RBD domain. As a result of this binding, a series of events occur, all of which assist in fusing the host cell and viral membranes for cell entrance. This interaction helps the spike protein transition from pre-metastable to post-metastable [55]. Recently, a new variant of concern, B.1.620, with 23 mutations in total, including S477N, E484K, D614G, and P681H, has been reported. This strain has gone unnoticed and has been spreading in Europe since February 2021. Initial reports revealed that it is a neutralizing antibody-escaping variant, but no concluding data are available to reveal the importance of this variant. Hence, a thorough investigation of the RBD-specific mutations, i.e., S477N and E484K, is required to reveal more information on this variant. For instance, a detailed analysis of the other variants (B.1.1.7, B.1.351, P.1, B.1.617, and B.1.618) using computational modeling and simulation approaches discovered that these mutations increase the binding affinity toward the host receptor and escape the antibody response [26,31,56]. To explore the role of these mutations and the impact on the binding of RBD with ACE2, we also employed molecular docking, hydrogen bonding network, and molecular dynamics simulation analyses to explore the variations in affinity and binding in comparison with the wild type. The structure of the spike RBD in the complex with ACE2 was retrieved from RCSB, and mutations were introduced into the sequence. The B.1.620 variant’s RBD structure was modeled using Modeller software. The structures were minimized and prepared. Figure 1A represents the multi-domain spike protein with domain organization; Figure 1B shows the interface residues of the ACE2–RBD complex; and Figure 1C represents the superimposed structure of the wild-type RBD and B.1.620 RBD, which revealed an RMSD difference of 0.841 Å. The two mutations in the RBD domain are also shown as sticks in panel C.

### 3.2. Interaction Energy and Hydrogen Bonding Network Analysis

To reveal the binding variations between the wild-type and B.1.620 RBD binding protein–protein, molecular docking was performed using HADDOCK. Proteins identify one another in a crowded environment and adhere to each other in a very precise way. This process entails proteins and other biological complexes diffusing across a densely populated environment before binding (docking) to their chosen protein partner in a structurally distinct and specific manner. This continually recurring process is absolutely astonishing, given the huge size of these macromolecules, their great structural variety, and the population concentration of the biomolecular habitat. Our present understanding of protein interaction principles is considerably better than it was previously, allowing us to develop more effective docking complexes [55]. This plays a significant role in understanding essential cellular functions. Thus, docking of the wild-type RBD with the ACE2 receptor revealed a docking score of −122.6 +/− 0.7, similar to that of previous studies [26,31,56]. However, the docking score for the B.1.620 RBD–ACE2 complex was reported to be −124.9 +/− 3.8, which is a higher docking score than that of the wild-type complex. Moreover, the vdW energy in both the wild-type and B.1.620 complexes remained comparable; however, marginal variations in the electrostatic energies were predicted. It can be seen that the electrostatic energy in the B.1.620 complex is −203.5 +/− 17.1, while in the wild type, it is −181.4 +/− 15.5, which suggests that these mutations increase the electrostatic energy. Similar findings are also reported by other studies on other variants [26,31,56]. However, similar docking results higher for the variants are reported in a recent report [16]. The docking scores, including the HADDOCK scores, vdW, electrostatic energy, and other parameters predicted by HADDOCK for both the complexes, are given in Table 1.

**Figure 2:**
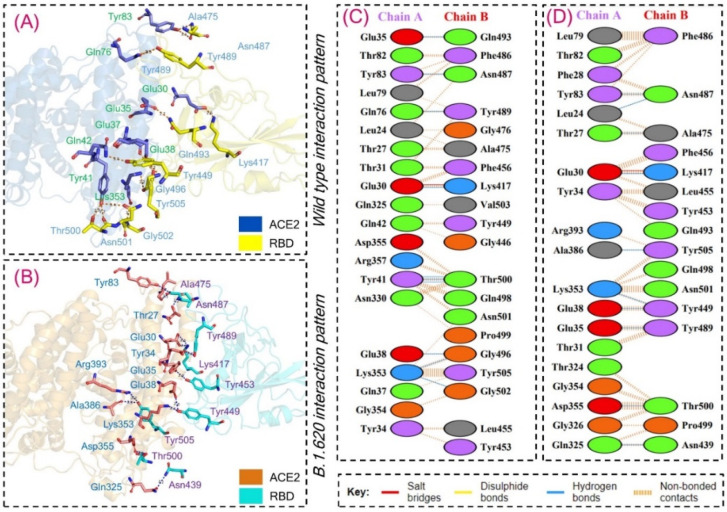
Interaction paradigm of the wild-type and B.1.620 RBD in complex with ACE2. Panel (**A**) represents the 3D interaction of the wild-type RBD with ACE2, (**B**) represents the 3D interaction of the B.1.620 RBD with ACE2, and (**C**,**D**) represent the 2D interaction of the wild-type and B.1.620 RBD with ACE2.

We further studied hydrogen bonding networks in detail to explore variations in the interaction pattern of each complex. Despite the variations in the HADDOCK energy of the wild-type and B.1.620 complexes, the total number of hydrogen bonds and salt bridges remained similar in both the complexes. Variations in the binding residues were detected, but the numbers remained similar. For instance, ten hydrogen bonds in each complex and a single salt bridge in both the complexes were detected. The Glu30–Lys417 interaction is strongly conserved in both complexes. Moreover, the Glu35–Gln493 interaction, which is required for correct orientation and locking, is only present in the wild-type complex while being absent in the B.1.620 complex. Lys353 forms an important cluster of interaction with multiple residues and was reported to interact with two residues, namely, Gly496 and Gly502, in the wild-type complex, while interacting with Tyr449 in the B.1.620 complex. Glu39–Tyr449 interaction is also important for the stabilized binding of RBD to ACE2 and is only reported in B.1.620 while being absent in the wild type. In both the complexes, the interaction between Tyr83 and Asn487 remained strongly conserved. Glu35 and Gln493 established hydrogen bonds with Glu38 and Gly496 in the wild-type complex, while these residues interacted with Tyr449 and Tyr489 in the B.1.620 complex. The Gln76–Tyr489 interaction was only detected in the wild-type complex. Moreover, Tyr41 formed three hydrogen bonds with Thr500 in the wild-type complex but not in the B.1.620 complex. Unique interactions only detected in the B.1.620 RBD–ACE2 complex but not in the wild-type complex include Leu24–Asn487, Thr27–Ala475, Gln325–Asn439, Asp355–Thr500, and Ala386–Tyr505 [26,31,56]. This shows that some interactions are preserved in the wild-type and B.1.620 complexes, while the specific variations in B.1.620 affect virus behavior and impact transmission. The only salt bridge in both the complexes, Glu30–Lys417, remained conserved and was reported by other studies on other variants [26,31,56]. For instance, the Tyr83–Asn487 interaction has been reported to distinguish the higher infectivity between SARS and SARS-CoV-2. The most important residue, Lys353, which interacts with many important residues, has also been reported to help in the binding and processing of RBD. Moreover, the Glu35 interactions with Tyr453 have been deemed as important interactions for the correct orientation locking of RBD to ACE2 [26,31,56]. The interaction patterns of the wild type and B.1.620 are shown in Figure 2A–D, while the interactions along with other details are given in Table 2.

### 3.3. Structural/Dynamic Features Investigation

#### 3.3.1. Dynamic Stability Calculation

**Figure 3:**
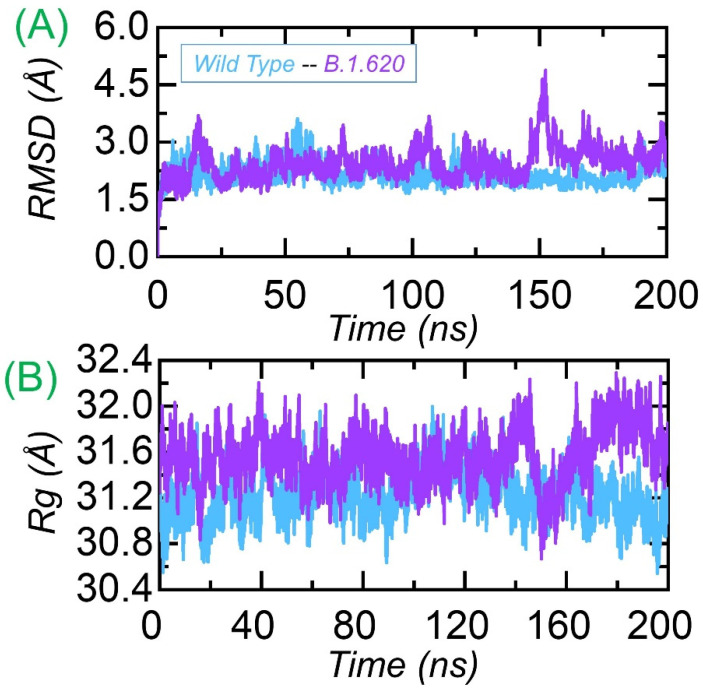
Dynamic stability and compactness of the wild-type and B.1.620 RBD in complex with ACE2. Panel (**A**) represents the RMSD(s) of the wild-type RBD and B.1.620 RBD with ACE2, and (**B**) represents RMSD(s) of the wild-type RBD and B.1.620 RBD with ACE2.

The structural/dynamic stability of the wild-type and B.1.620 complexes was estimated as the root mean square deviation (RMSD) as a function of time. The wild-type and B.1.620 complexes were subjected to RMSD analysis over the 200 ns simulation trajectory. The wild-type complex remained more stable compared to the B.1.620 complex. The average RMSD for the wild-type complex was reported to be 2.0 Å. Smaller deviations were observed during the first 75 ns, but then the RMSD stabilized, and no deviation was reported during the last 125 ns. In the case of B.1.620, the complex reported significant deviations at different time intervals over the simulation time. During the first 25 ns, significant deviation was observed, particularly between 15 and 25 ns. Then, the RMSD stabilized for some time, 26–75 ns, and remained lower than the first 25 ns. The RMSD then converged between 75 and 78 ns, and then again, the RMSD stabilized. The RMSD experienced convergence between 100 and 120 ns and then again converged. The B.1.620 complex exhibited significant deviation for 150–175 ns. The structure then stabilized until 200 ns, and the average RMSD was observed to be ~2.5 Å. The previous literature reported that mutations that induce destabilizing effects produce a radical function; thus, the behavior of B.1.620 is more detrimental than that of the wild type [11,31]. However, a particular mutation in the RBD, C432D, lessens ACE2-assisted entry into the cell using a spike trimer. The findings strongly corroborate previous findings, where mutations with stability induction correlate with a higher binding affinity [41]. Consequently, we reported that the higher binding of B.1.620 with a destabilizing effect has a radical function and, thus, increases infectivity. The RMSDs of the wild-type and B.1.620 complexes are shown in Figure 3A.

#### 3.3.2. Structural Compactness Analysis

Understanding the structural compactness in a dynamic environment using simulation helps in understanding the packing of protein–protein complexes and demonstrates the binding and unbinding events that occur during the simulation. We calculated structural compactness as the radius of gyration (Rg) using the simulation trajectories. In Figure 3B, it can be seen that the wild type remained more compact than B.1.620. Though the structural compactness of B.1.620 during the first 155 ns remained uniform, the Rg decreased to 30.8 Å and then increased again. This shows the binding and unbinding events and more conformational optimization for binding, which corroborates previous findings [26,31,56]. The Rg(s) for each complex is shown in Figure 3B.

**Figure 4:**
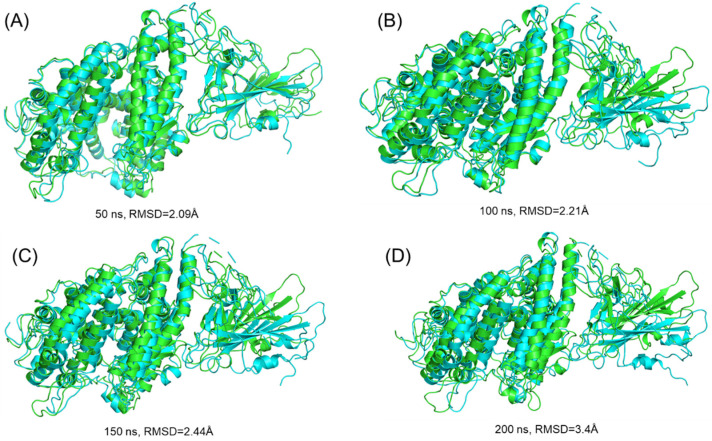
Structural superimposition analysis of the wild-type and B.1.620 variant at different time intervals ((**A**–**D**) at 50, 100, 150, and 200 ns, respectively).

We further extracted the structures of both wild-type and B.1.620 complexes at 50, 100, 150, and 200 ns to see the RMSD differences by superimposing them on each other. As presented in Figure 4, the RMSD at 50 ns was 2.09 Å, at 100 ns it was 2.21 Å, and at 150 ns it was 2.44 Å, while at 200 ns, a 3.40 Å RMSD difference was observed. This shows the structural and dynamic variations induced by the mutations, affecting the dynamic properties.

#### 3.3.3. Residual Flexibility Analysis

**Figure 5:**
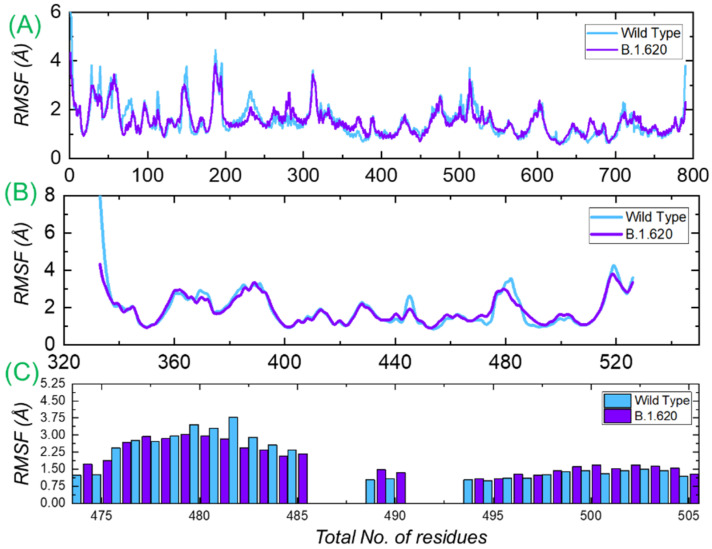
Residual flexibility of the wild-type and B.1.620 RBD in complex with ACE2. Panel (**A**) represents the RMSF of the wild-type RBD and B.1.620 RBD with ACE2, and (**B**) represents RMSF(s) of the wild-type RBD and B.1.620 RBD apo. Panel (**C**) represents the RMSF of the three loops required for interaction.

Residual flexibility is always an important parameter for exploring the binding strength induced by a particular residue. It is the main factor that determines the strength of the binding of biological molecules. To determine the flexibility of each residue, we calculated the root mean square fluctuation (RMSF) for each complex. It can be seen that the RMSF of the wild-type and B.1.620 complexes is primarily comparable, and at different regions, the wild type displayed a relatively higher fluctuation. The RMSF of the B.1.620 complex is stabilized by the binding of the interacting proteins and, thus, displays minimal fluctuation. Figure 5A shows the RMSF of the wild-type and B.1.620 RBD in complex with ACE2. We further calculated the RMSD of the apo RBD of both the wild type and B.1.620. Both the complexes displayed similar fluctuation except in the regions between 475 and 490, where mutations lie (Figure 5B). Moreover, to see the flexibility profile of the three important loops (474–485, 488–490, and 494–505) previously reported to be the most important residues for the interaction, we calculated the RMSFs for these loops, which are presented in Figure 5C. The structure shows that some of the fluctuations are stabilized while some are conserved, as previously reported [26].

#### 3.3.4. Hydrogen Bonding Analysis

**Figure 6:**
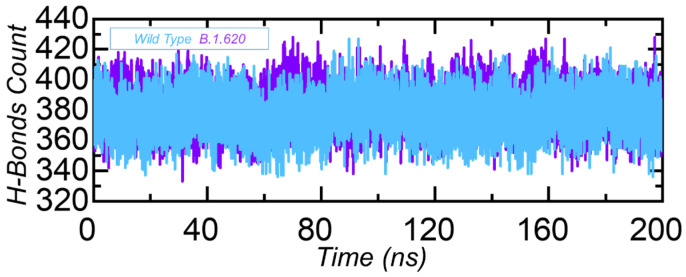
Hydrogen bond count in the wild-type RBD and B.1.620 RBD in complex with ACE2. The x-axis shows time in nanoseconds, while the y-axis shows hydrogen bond count in each complex.

Protein–protein interactions are primarily influenced by a number of different factors, the most important of which are the hydrogen bonds and hydrophobic interactions. Water molecules constantly occupy protein interfaces, competing with hydrogen bonding among residues [57]. The mechanisms underlying protein–protein complex formation, as well as the ramifications of hydrogen bonds’ participation in this relationship, are unclear [58]. The question of whether or not hydrogen bonds regulate protein–protein binding is a long-standing one with a process that remains poorly understood [59,60]. Thus, to reveal variations in the bonding pattern between the wild-type RBD and B.1.620 RBD complexes, we performed a hydrogen bonding network analysis of the simulation trajectory to demonstrate the binding specificity for each complex steered by hydrogen bonding. The calculation of the total hydrogen bonding revealed that the wild-type complex has, on average, 377 hydrogen bonds, while the B.1.620 complex has 383 hydrogen bonds. For the other variants, such as B.1.1.7, B.1.351, B.1.617, B.1.618, and P.1, the higher number of hydrogen bonds are already reported, which strongly justify our findings [26,31,56]. This shows that significant reprogramming of the hydrogen bonds took place during the simulation, thus increasing binding affinity. The total number of hydrogen bonds in each complex is shown in Figure 6.

#### 3.3.5. Solvent Accessible Surface Area (SASA)

**Figure 7:**
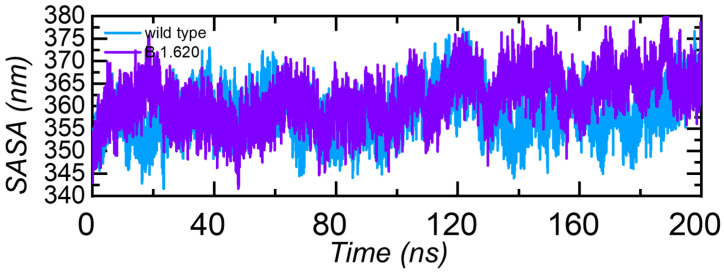
Solvent accessible surface area (SASA) of the wild-type RBD and B.1.620 RBD in complex with ACE2. The x-axis shows time in nanoseconds, while y-axis shows SAAS in nm^2^ in each complex.

Solvent accessible surface area is another important measure that calculates the area accessible to the solvent molecule. The SASA of the B.1.620 complex is higher than that of the wild-type complex (Figure 7). Increased SASA values indicate the relative expansion of mutant structures and increased intrinsic flexibility, which increases the likelihood of stable binding of the interacting protein with ACE2.

### 3.4. Binding Free Energy Calculation

The approximation of the binding free energy of two biomolecules governs the strength of the bio-molecular complex. The current computational valuation of the free energy by the MM/GBSA method is the most widely employed approach to re-rank the docking complex by forecasting the dynamic stability and strength of key binding hotspots and the total binding energy. The aforementioned method is computationally inexpensive compared to the substitute methods, i.e., alchemical free energy calculation approaches. The MM/GBSA technique is considered as more accurate and precise than the conventional scoring algorithms [61]. Seeing the higher applicability of the specified method, we projected the impact of new substitutions S477N-E484K in the RBD on the binding to the ACE2 receptor. The total binding free energy results given in Table 3 show that the B.1.620 RBD–ACE2 complex has a higher binding affinity than that of the wild-type complex. The vdWs for these two complexes were reported to be −107.63 kcal/mol and −100.46 kcal/mol respectively, while the electrostatic energy was increased in the B.1.620 RBD–ACE2 complex. These findings are in accordance with those of previous reports, where a higher electrostatic energy for the variants was reported to be the main factor that contributes to the higher binding affinity [26,31,56]. Thus, this shows that our findings strongly correlate with the previous findings. The electrostatic energy for each complex was reported to be −592.87 kcal/mol for the wild type and −1129.31 kcal/mol for B.1.620. The total binding energy for the wild type was −51.14 kcal/mol, while for B.1.620, the total binding energy was −68.75 kcal/mol. Moreover, we also calculated the binding free energy by using the MM/PBSA approach, which revealed that both MM/GBSA and MM/PBSA strongly correlate with each other in terms of defining the final total energy. It can be seen, as given in Table 3, that the wild type has less binding free energy than that of the B.1.620 variant. Furthermore, here, the notion of more electrostatic energy can also be seen to have a higher contribution than the other factors. It can be observed that the MM/PBSA total binding energy for the wild type is −16.54 kcal/mol, while for B.1.620, it is −22.32 kcal/mol. Consequently, this shows that the specific mutations in the RBD (S477N-E484K) help the new variant to increase binding affinity and, consequently, infectivity.

## 4. Conclusions

The pandemic caused by SAR-COV-2 has been further exasperated by the evolution of new variants reported since 2020. The new variants are reported to have mutations in the spike protein, which affect binding and infectivity. Despite the growing interest in COVID-19 research, a comprehensive evaluation of the illness is required. SARS-CoV-2, a severe acute respiratory CoV, with improved virus–host dynamics in terms of improved binding, may have resulted in increased pathogenicity. As a result, a better knowledge of the viral mutations and evolution is required. Although just a few variant sites for SARS-CoV-2 variants have been discovered, the range of intra-host variant contacts and dynamics linked to the virus’s progression is unclear. The current findings based on protein complex modeling and bio-simulation methods revealed the atomic features of the B.1.620 variant harboring S477N and E484K mutations in the RBD. Understanding the interaction dynamics is key to the protein–protein recognition process and, consequently, the regulation of important cellular functions. A study on more polymorphic sites to understand the evolution pattern and the prediction of emerging variants may help to contain this pandemic. Large-scale genomic analyses are needed to decipher the mutational spectrum of SARS-CoV-2 at a genomic scale, and then connecting these patterns to the genomic attributes could potentially control the evolution of this virus. Our analysis revealed that though the number of hydrogen bonds and salt bridges remained the same, the binding affinity of B.1.620 for ACE2 was higher than that of the wild type, consequently increasing infectivity. These features can be used to design drugs that could efficiently inhibit the interaction of the RBD with ACE2.

## Figures and Tables

**Table 1:** HADDOCK-predicted docking score for the wild-type RBD–ACE2 and B.1.620 RBD–ACE2 complexes.

Parameters	Wild-Type RBD–ACE2 Complex	B.1.620 RBD–ACE2 Complex
HADDOCK scores	−122.6+/− 0.7	−124.9 +/− 3.8
Cluster size	64	20
RMSD in Å	1.7 +/− 1.0	14.3 +/− 0.2
vdW	−59.6 +/− 2.3	−59.4 +/− 4.4
Electrostatic energy	−181.4 +/− 15.5	−203.5 +/− 17.1
Desolvation energy	−27.1 +/− 3.4	−25.6 +/− 2.1
Restraint’s violation energy	4.7 +/− 3.8	5.4 +/− 1.9
Buried surface area (A^2^)	1965.3 +/− 120.6	1906.8 +/− 52.5
Z-score	−1.9	−1.1

**Table 2:** Interaction paradigm, including hydrogen bonds and salt bridges of the wild-type and B.1.620 RBD in complex with ACE2. The table also represents the bond distance in Å.

Complex Name	ACE2 Interacting Residues	RBD Interacting Residues	Distance (Å)	Type of Bond
Wild Type	GLU30	LYS417	2.56	Hydrogen Bond
	GLU35	GLN493	2.75	Hydrogen Bond
	GLU38	GLY496	3.16	Hydrogen Bond
	TYR41	THR500	2.73	Hydrogen Bond
	TYR41	THR500	2.68	Hydrogen Bond
	TYR41	THR500	2.68	Hydrogen Bond
	GLN76	TYR489	3.06	Hydrogen Bond
	TYR83	ASN487	2.73	Hydrogen Bond
	LYS353	GLY502	3.15	Hydrogen Bond
	LYS353	GLY496	3.16	Hydrogen Bond
	GLU30	LYS417	2.56	Salt Bridge
B.1.620	LEU24	ASN487	3.08	Hydrogen Bond
	THR27	ALA475	3.23	Hydrogen Bond
	GLU30	LYS417	2.55	Hydrogen Bond
	GLU35	TYR489	2.65	Hydrogen Bond
	GLU38	TYR449	2.61	Hydrogen Bond
	TYR83	ASN487	2.81	Hydrogen Bond
	GLN325	ASN439	3.05	Hydrogen Bond
	LYS353	TYR449	2.74	Hydrogen Bond
	ASP355	THR500	2.92	Hydrogen Bond
	ALA386	TYR505	3.31	Hydrogen Bond
	GLU30	LYS417	2.55	Salt Bridge

**Table 3:** Binding free energy calculation for the wild-type and B.1.620 complexes calculated as MM/GBSA and MM/PBSA. All the energies are given in kcal/mol.

MM/GBSA	VDW	ELE	GB	SA	Total
Wild Type	−107.63	−592.87	663.18	−13.82	−51.14
B.1.620	−100.46	−1129.31	1174.64	−13.62	−68.75
**MM/PBSA**	**VDW**	**ELE**	**PB**	**ESURF**	**Total**
Wild Type	−52.21	−76.42	118.74	−6.65	−16.54
B.1.620	−54.27	−85.35	126.37	−9.07	−22.32

## Data Availability

The data presented in this study are available within the article.
